# Selenium, Selenoenzymes, Oxidative Stress and Risk of Neoplastic Progression from Barrett's Esophagus: Results from Biomarkers and Genetic Variants

**DOI:** 10.1371/journal.pone.0038612

**Published:** 2012-06-08

**Authors:** Yumie Takata, Alan R. Kristal, Regina M. Santella, Irena B. King, David J. Duggan, Johanna W. Lampe, Margaret P. Rayman, Patricia L. Blount, Brian J. Reid, Thomas L. Vaughan, Ulrike Peters

**Affiliations:** 1 Public Health Science Division, Fred Hutchinson Cancer Research Center, Seattle, Washington, United States of America; 2 Department of Environmental Health Sciences, Columbia University, New York, New York, United States of America; 3 Department of Internal Medicine, University of New Mexico, Albuquerque, New Mexico, United States of America; 4 Division of Genetic Basis of Human Disease, Translational Genomics Research Institute, Phoenix, Arizona, United States of America; 5 Faculty of Health and Medical Sciences, University of Surrey, Guildford, United Kingdom; 6 Human Biology Division, Fred Hutchinson Cancer Research Center, Seattle, Washington, United States of America; Vanderbilt University Medical Center, United States of America

## Abstract

Clinical trials have suggested a protective effect of selenium supplementation on the risk of esophageal cancer, which may be mediated through the antioxidant activity of selenoenzymes. We investigated whether serum selenium concentrations, selenoenzyme activity, oxidative stress and genetic variation in selenoenzymes were associated with the risk of neoplastic progression to esophageal adenocarcinoma (EA) and two intermediate endpoints, aneuploidy and tetraploidy. In this prospective cohort study, during an average follow-up of 7.3 years, 47 EA cases, 41 aneuploidy cases and 51 tetraploidy cases accrued among 361 participants from the Seattle Barrett's Esophagus Research Study who were free of EA at the time of blood draw and had at least one follow-up visit. Development to EA was assessed histologically and aneuploidy and tetraploidy by DNA content flow cytometry. Serum selenium concentrations were measured using atomic absorption spectrometry, activity of glutathione peroxidase (GPX) 1 and GPX3 by substrate-specific coupled test procedures, selenoprotein P (SEPP1) concentrations and protein carbonyl content by ELISA method and malondialdehyde concentrations by HPLC. Genetic variants in *GPX1-4* and *SEPP1* were genotyped. Serum selenium was not associated with the risk of neoplastic progression to EA, aneuploidy or tetraploidy (P for trend = 0.25 to 0.85). SEPP1 concentrations were positively associated with the risk of EA [hazard ratio (HR) = 3.95, 95% confidence intervals (CI) = 1.42–10.97 comparing the third tertile with the first] and with aneuploidy (HR = 6.53, 95% CI = 1.31–32.58), but not selenoenzyme activity or oxidative stress markers. No genetic variants, overall, were associated with the risk of neoplastic progression to EA (global p = 0.12–0.69). Our results do not support a protective effect of selenium on risk of neoplastic progression to EA. Our study is the first to report positive associations of plasma SEPP1 concentrations with the risk of EA and aneuploidy, which warrants further investigation.

## Introduction

Results from a few clinical trials and observational studies support a protective effect of selenium on the risk of esophageal cancer [1]–[5]. Two trials in China found non-significant reduced risks of esophageal cancer (primarily squamous cell carcinoma) with supplementation of selenium in combination with other micronutrients for more than five years [1]–[3]. Case-cohort analysis within one of these trials found a statistically significant 44% lower risk of esophageal cancer among supplemented participants whose pre-trial serum selenium concentrations were in the highest (>82 µg/L) rather than the lowest (<60 µg/L) quartile [4]. In the U.S., secondary analysis of the Nutritional Prevention of Cancer Trial showed a non-significant 70% lower risk of esophageal cancer in the group supplemented with selenium alone than in the placebo group after 6.4 years of follow-up, but included only eight esophageal cancer cases [5]. Further, two small case-control studies showed an inverse association between serum selenium concentrations and the risk of esophageal cancer, but the lower selenium status observed may have been caused by the disease [6], [7]. The only previous study of genetic variants in selenoenzymes and risk of esophageal adenocarcinoma (EA) reported null finding [8].

Here we investigated the association of serum selenium with the risk of neoplastic progression to EA. Persons with Barrett's esophagus (BE), a premalignant metaplasia of the lower esophageal epithelium, develop EA at a rate of 6–7 per 1,000 person-years, which is substantially higher than in the general population [9]. Thus, this cohort provides an opportunity to investigate longitudinally the neoplastic progression to EA, which is a very rare cancer in the general population. In a previous cross-sectional analysis of BE patients in this cohort, we observed an inverse association between serum selenium concentration and various markers of neoplastic progression to EA [10]. This study further investigates these results, by longitudinally examining the associations of selenium, selenoenzymes/selenoproteins and oxidative stress with subsequent risk of neoplastic progression to EA, aneuploidy, and tetraploidy. Because selenium exerts its antioxidant property through selenoenzymes, we also examined whether the activity of, as well as genetic variations in, selenoenzymes were associated with the risk of progression.

## Methods

### Study population

This prospective cohort study was conducted through the Seattle Barrett's Esophagus Research Study, a dynamic cohort of persons diagnosed with BE [11]. The study was initially approved by the Human Subjects Division of the University of Washington in 1983 and was renewed annually thereafter with reciprocity from the Fred Hutchinson Cancer Research Center (FHCRC) from 1993 to 2001. Since 2001, the study has been approved by the Institutional Review Board of the FHCRC with reciprocity from the Human Subjects Division of the University of Washington. All participants provided written informed consent. The study began in 1983 with endoscopic surveillance and was expanded as of February 1, 1995 to include the collection of blood, interview and anthropometric data. Participants underwent an extensive baseline interview, after which they had shorter follow-up interviews at subsequent endoscopies. The structured baseline interview took approximately 45 minutes to complete and collected detailed information on various lifestyle exposures, medical and medication history, and anthropometric measurements (height, weight, and circumference of waist, hips, thighs, and abdomen). At follow-up interviews, updated information was obtained on anthropometric measurements, lifestyle and current medication use, and a blood sample was collected.

Endoscopic biopsy protocols and evaluations used in the Seattle Barrett's Esophagus Research Study have been published previously [11]–[13]. Briefly, for those without high-grade dysplasia, four-quadrant biopsies were taken for histologic evaluation every two centimeter throughout the length of the Barrett's segment; for those with a history of high-grade dysplasia, every one centimeter throughout the length of the Barrett's segment. Persons entering the cohort with an outside diagnosis of high-grade dysplasia were further evaluated by an intensive protocol of four-quadrant biopsies every one centimeter two additional times within the first four months of participation, after which they were followed up approximately every six months. Those without high-grade dysplasia or a history of high-grade dysplasia were followed up every two to three years. Participants' histology was classified according to the highest grade of dysplasia present and study pathologists were blinded to participant information. Overnight fasting blood samples were collected prior to endoscopy and serum and EDTA-treated plasma aliquots were prepared and stored at −70°C.

A total of 361 participants free of EA at the time of blood draw and with at least one follow-up visit were included in analyses based on serum selenium. For the analysis for aneuploidy and tetraploidy, 37 and 36 participants, respectively, were excluded from the analysis due to the diagnosis of the respective endpoint at the time of blood draw. As a result, analyses for EA, aneuploidy and tetraploidy had 2647.2, 2378.2 and 2290.0 person-years, respectively. For the subset of 198 participants who had given sufficient blood at the first or second follow-up visit for the laboratory assays, selenoenzyme activity, or selenoprotein concentration and two measures of oxidative stress were made. In addition, single nucleotide polymorphisms (SNPs) in selenoenzyme/selenoproteins were genotyped in this subset.

### Outcome measures

EA was assessed histologically, and aneuploidy and tetraploidy were assessed as previously described [14]. Briefly, DNA content was measured by flow cytometry using the computer program Multicycle (Phoenix Flow Systems, San Diego, CA) [13], [15]. A diagnosis of aneuploidy was made if discrete peaks on the histogram showed aneuploid and diploid cell populations and if the aneuploid peak included at least 2.5% of cells in the biopsy sample [13]. A diagnosis of tetraploidy (4N) was made if >6% of cells had DNA content between 3.85 and 4.10 N.

### Laboratory measures

Serum selenium concentrations were measured by atomic absorption spectrometry (Perkin Elmer, Fremont CA) according to the standard protocol [16]. This was conducted at the Harborview Medical Center and the FHCRC. To ensure the comparability of the assays conducted at the two locations, 57 samples were run at both locations, yielding a mean coefficient of variation (CV) of 6.0% (range: 0.24–18.4%, pair: 57). The overall mean CV from blinded duplicate samples run at either or both locations was 15.1% (range: 0.24–39.0%, pair: 72). For a large fraction of eligible participants, selenium was measured at baseline and follow-up visits, yielding a total of 647 values for 369 participants. Due to the high CVs (>10%) observed in two pairs, we excluded 82 selenium values that were analyzed on the same day as these pairs. After the exclusion, the CV (mean ± standard deviation) of selenium values from the same participants measured in blood samples collected at different time points was 8.8±6.4%. Hence, among the remaining selenium values, the analysis included the blood sample that was collected during the earliest study visits to maximize the time to event. Included in the analysis were 349 samples from baseline, eleven from first follow-up and one from the second follow-up.

The activity of glutathione peroxidase (GPX) 1 in white blood cells and of GPX3 in plasma were measured by applying our standardized protocol using OXItek commercial kit [ZMC catalog # 0805002, ZeptoMetric Corporation, Buffalo NY,] based on Paglia and Valentine's method [17] and using cumene hydroperoxide as the substrate. Quality controls (QCs) of known activity were run at the beginning of the assay each day to ensure the quality of assay internally. The respective mean CV of GPX1 and GPX3 activity from all samples run as duplicates was 2.1% and 3.2%. Both GPX1 and GPX3 assays were conducted at FHCRC. Selenoprotein P (SEPP1) concentration was measured in plasma samples using a sandwich ELISA method as previously described [18]. N22 was used as a capture antibody and N11 as a biotinylated antibody since each antibody recognizes different proportions of the N terminus of the human protein. The CV (mean ± standard deviation) of blinded QCs from two plasma samples, each measured seven times, was 6.8% (4.83±0.33 µg/L) and 17.1% (5.14±0.88 µg/L). The SEPP1 assay was conducted at the Vanderbilt University School of Medicine.

To assess lipid peroxidation, malondialdehyde (MDA) was assayed in EDTA-treated plasma using HPLC as previously described [19]. This assay was conducted at FHCRC. For the assessment of protein oxidation, protein carbonyl content (PCC) in plasma was analyzed by the non-competitive ELISA method using dinitrophenylhydrazine as an antibody that was developed previously [20]. QCs with known PCC were included in each plate. The CV (mean ± standard deviation) from internal QCs in all six plates was 10.0% (0.40±0.04 µmol/L) and the CVs of blinded QCs from two plasma samples measured each seven times were 16.1% (0.37±5.96 µmol/L) and 12.8% (0.39±5.01 µmol/L). This assay was conducted at the Columbia University.

A set of tagging SNPs (tagSNPs) in each of five selenoenzyme/selenoprotein genes (i.e., *GPX1*, *GPX2*, *GPX3*, *GPX4* and *SEPP1*) was selected because these genes are associated with oxidative stress and expressed in the gastrointestinal tract [21]–[23]. We first sequenced these genes in European American subjects in the HapMap population [24] and selected all SNPs in the selenocysteine insertion sequence and all nonsynonymous SNPs in exons [24]. Secondly, additional tagSNPs were selected according to the criteria of *r* ^2^≥0.8 and minor allele frequency ≥5% [25] based on our sequencing data [24] on European American HapMap samples [26]. A total of 34 tagSNPs were genotyped using Matrix-assisted Laser Desorption/Ionization Time-of-Flight on the Sequenom MassARRAY 7K platform (Sequenom, Inc., San Diego CA) at the Translational Genomics Research Institute. Each plate included blinded duplicates from 5% of study samples as QC. Twelve SNPs were excluded for the following reasons: call rate <90% for three SNPs (rs75404373, rs2277501 and rs4807542); P-value for Hardy-Weinberg equilibrium <0.01 for four SNPs (rs2293627, rs6888691, rs3763011 and rs757229); concordance of the blinded QCs (10 pairs) <90% for two SNPs (rs2074452 and rs7579) and minor allele frequency ≤5% for three SNPs (rs8179164, rs17883875 and rs4807543).

### Statistical Analyses

Cox proportional hazards regression models were used to assess the association of selenium, selenoenzyme activity, selenoprotein concentrations, oxidative stress and genetic variants in selenoenzymes with time to neoplastic progression to EA, aneuploidy and tetraploidy. The time of entry was time of the blood draw for selenium, selenoenzyme and oxidative stress marker analyses and time of baseline interview for genetic analysis. The time of exit was defined as the date of endpoint diagnosis or date of the last follow-up visit as of September 31, 2009, whichever came first. Serum selenium concentration was analyzed as continuous, grouped into tertiles based on the distribution in the entire cohort, and classified into low and high concentrations based on the cut-off of 118 µg/L used in our previous study (defined as the 75th percentile cut-off) [10]. For MDA concentrations, nine outliers defined as being outside of the upper and lower three interquartile ranges were excluded. GPX1 activity and MDA concentrations were log-transformed. Selenoenzyme activity and selenoprotein concentration as well as oxidative stress markers were categorized by tertile. Tests for linear trend across tertiles were based on median values in each tertile.

All analyses were controlled for gender, baseline values of age (5 categories), waist to hip ratio (quartiles), smoking status (never, former or current) and the use of non-steroid anti-inflammatory drugs (NSAIDs; never, former or current). Genetic analysis was also controlled for Caucasian ethnicity (European American or other). All covariates each affected the hazard ratio (HR) of at least one of the outcomes by a minimum of 10%.

For genetic analysis of five selenoenzyme/selenoprotein genes, the overall variation within a gene was first evaluated by global gene test, which compared the log likelihood ratio statistics between the model with and without all SNPs in a given gene [27]. If the test was significant (P<0.05), significant association of individual SNPs was considered. The number of the minor allele was coded as log-additive (i.e., 0 for homozygote common allele, 1 for heterozygotes and 2 for homozygote rare allele) to test for trend. The effect of individual SNPs was tested by including one SNP per model.

To assess if prevalence of high-grade dysplasia, aneuploidy or tetraploidy influenced the results, a sensitivity analysis was conducted in two ways: 1) excluding participants with high-grade dysplasia and 2) excluding those with aneuploidy and/or tetraploidy at baseline and considering two outcomes (EA and aneuploidy and/or tetraploidy). The results from the sensitivity analysis were compared with those from the overall analyses. All statistical analyses were conducted by SAS 9.2 (Carey, NC) and STATA 11 (College Station, TX).

## Results

The majority of study participants were Caucasian and male (**Table 1**), reflecting the typical distribution of these characteristics in BE patients. More than half of the participants had at least some college education. Participants who developed EA, aneuploidy or tetraploidy tended to use NSAIDs less often than those who did not. EA cases had smoked substantially more than aneuploidy or tetraploidy cases or the total cohort. The proton pump inhibitor or H2 blocker medication was used by almost all participants who had not gone through anti-reflux surgery. All participants had gastroesophageal reflux disease symptoms prior to or at the time of the study enrollment. EA cases were most likely to have high-grade dysplasia at baseline, followed by cases with aneuploidy and tetraploidy. At baseline, both aneuploidy and tetraploidy were common among EA cases compared with the overall cohort or tetraploidy or aneuploidy cases. Among the study participants, there were 33 participants who developed high-grade dysplasia, but not EA during the study period. There were 10 participants who had all three conditions (aneuploidy, tetraploidy and high-grade dysplasia) at baseline. There was a broad range in serum selenium concentrations, which varied from 67.1 to 213.2 µg/L.

**Table 1:** Characteristics of the Study Population*.

	All	Esophageal adenocarcinoma	Aneuploidy	Tetraploidy
Number	361	47	41	51
Women	68 (18.8%)	6 (12.8%)	13 (14.6%)	10 (19.6%)
Age at baseline	61.1±11.7	64.2±10.6	61.0±10.7	63.4±11.6
Age at last follow-up visit	68.4±11.5	67.5±10.7	67.4±10.0	71.1±10.7
Waist to Hip ratio at baseline	0.95±0.07	0.96±0.07	0.96±0.07	0.96±0.08
Ethnicity				
Caucasian	337 (93.4%)	46 (97.9%)	39 (95.1%)	47 (92.2%)
Others	24 (6.6%)	1 (2.1%)	0 (0%)	1 (2.0%)
Educational attainment				
Grade school	6 (1.7%)	2 (4.3%)	0 (0%)	1 (2.0%)
High school	91 (25.3%)	14 (29.8%)	12 (33.3%)	16 (31.4%)
Technical/vocational school	21 (5.8%)	4 (8.5%)	1 (8.1%)	4 (7.8%)
College	242 (67.2%)	27 (57.4%)	28 (55.6%)	30 (58.8%)
NSAID use at baseline				
Cumulative use (pill-months)	75.9±163.5	61.1±131.8	61.7±120.1	59.8±101.6
Never	149 (41.6%)	24 (51.1%)	21 (51.2%)	20 (39.2%)
Former	74 (20.7%)	10 (21.3%)	10 (24.4%)	11 (21.6%)
Current	135 (37.7%)	13 (27.6%)	10 (24.4%)	20 (39.2%)
Smoking at baseline				
Pack-years	18.8±24.0	25. 8±24.0	15.4±16.7	17.6±21.8
Never	127 (35.2%)	10 (21.3%)	13 (31.7%)	17 (33.3%)
Former	197 (54.6%)	35 (74.5%)	24 (58.5%)	32 (62.7%)
Current	37 (10.2%)	2 (4.2%)	4 (9.8%)	2 (3.9%)
Serum selenium concentration (µg/L)	135.0±21.0	136.2±19.4	138.7±20.0	136.9±19.3
GPX1 activity (U/g T protein)**	43.1±21.8	45.6±20.0	41.3±12.3	43.0±16.5
GPX3 activity (U/L)**	729±121	704±134	699±111	691±126
SEPP concentration (µg/L)**	5.78±1.12	6.25±1.12	5.88±1.09	5.78±1.27
Malondialdehyde (µmol/L)**	1.09±1.15	1.26±1.29	1.06±1.00	1.06±0.95
Protein carbonyl content (nmol/mg protein)**	0.36±0.06	0.36±0.06	0.35±0.06	0.37±0.06
High-grade dysplasia at baseline	66 (18.3%)	34 (72.3%)	46 (46.3%)	19 (37.3%)
Aneuploidy at baseline	37 (10.2%)	19 (40.0%)	0 (0%)	24 (47.1%)
Tetraploidy at baseline	36 (10.0%)	19 (40.0%)	13 (31.7%)	0 (0%)

* The mean ± standard deviation or number (percentage) is provided.

** Up to 198 of the participants were included.

Serum selenium concentrations were not associated with the risk of neoplastic progression to EA (**Table 2**). The adjusted HRs [95% confidence intervals (95% CI)] for EA, aneuploidy and tetraploidy with each 50 µg/L increase in serum selenium concentrations were 1.16 (0.60–2.28), 1.64 (0.79–3.42) and 1.06 (0.54–2.06), respectively. Analysis based on tertiles of selenium concentrations also showed no association; the corresponding P for trend for EA, aneuploidy and tetraploidy were 0.39, 0.25 and 0.85. Compared with the participants with serum selenium concentration <118 µg/L, the concentration ≥118 µg/L was not associated with risk of neoplastic progression to EA (EA: HR = 1.23, 95% CI = 0.58–2.60; aneuploidy: HR = 2.15, 95% CI = 0.83–5.58; tetraploidy: HR = 1.38, 95% CI = 0.63–3.00, respectively). Excluding participants with high-grade dysplasia or aneuploidy and/or tetraploidy at baseline did not materially change results (data not shown).

**Table 2:** Association between Serum Selenium and the Risk of Neoplastic Progression to Esophageal Adenocarcinoma.

		Esophageal adenocarcinoma		Aneuploidy		Tetraploidy
Number of cases/all		47/361		41/324		51/325
	cases	HR (95% CI)*	cases	HR (95% CI)*	cases	HR (95% CI)*
**Serum selenium concentration**						
Per 50 µg/L	47	1.16 (0.60–2.28)	41	1.64 (0.79–3.42)	51	1.06 (0.54–2.06)
Tertile 1 (<126.3 µg/L)	12	reference	11	reference	14	reference
Tertile 2 (126.3–143.8 µg/L)	19	1.67 (0.79–3.53)	14	1.17 (0.51–2.66)	20	1.39 (0.37–2.88)
Tertile 3 (>143.8 µg/L)	16	1.40 (0.65–3.02)	16	1.60 (0.72–3.55)	17	1.60 (0.52–2.29)
*P for trend* **		*0.39*		*0.25*		*0.85*
<118 µg/L	9	reference	5	reference	8	reference
≥118 µg/L	38	1.23 (0.58–2.60)	36	2.15 (0.83–5.58)	43	1.38 (0.63–3.00)

* HRs were adjusted for age at time of blood draw (5 categories), waist: hip ratio (quartiles) at baseline, sex, smoking status and NSAID use (both for never, former or current).

** P for trend was obtained by assigning median values of each tertile.

SEPP1 concentrations were positively associated with the risk of EA and aneuploidy, but not tetraploidy (**Table 3**). Each one unit (µg/L) increase in SEPP1 concentration was associated with 46% increase in EA risk (HR = 1.46, 95% CI = 1.05–2.05). Those in the highest tertile of SEPP1 concentrations had a 3.95-time higher risk of EA (HR = 3.95, 95% CI = 1.42–10.97, P for trend = 0.006) and a 6.53-time higher risk of aneuploidy (HR = 6.53, 95% CI = 1.31–32.58, P for trend = 0.02) compared with those at the lowest. None of the other selenoenzymes (GPX1 and GPX3) or oxidative stress markers (i.e., MDA and PCC) was associated with the risk of neoplastic progression to EA. Excluding participants with high-grade dysplasia or aneuploidy and/or tetraploidy at baseline did not alter the observed positive associations of SEPP1 concentrations with the risk of EA and aneuploidy, which lost statistical significance, but were in the same direction (data not shown). We further examined the cross-sectional association of SEPP1 concentrations and the risk of high-grade dysplasia, aneuploidy or tetraploidy and found no association [high-grade dysplasia: odds ratio (OR) = 0.99, 95% CI = 0.70–1.40; aneuploidy: OR = 0.76, 95% CI = 0.49–1.16; tetraploidy: OR = 1.05, 95% CI = 0.73–1.53].

**Table 3:** Association of Selenoenzyme Activity or Concentration and Oxidative Stress with the Risk of Neoplastic Progression to Esophageal Adenocarcinoma.

		Esophageal adenocarcinoma		Aneuploidy		Tetraploidy
Number of cases/all		27/171		16/140		17/137
	cases	HR (95% CI)*	cases	HR (95% CI)*	cases	HR (95% CI)*
**GPX1 activity**						
Per U/T g protein	27	1.26 (0.50–3.17)	16	1.80 (0.47–6.80)	17	0.69 (0.20–2.37)
Tertile 1 (<35.2 U/T g protein)	7	reference	5	reference	5	reference
Tertile 2 (35.2–44.5 U/T g protein)	9	1.09 (0.38–3.11)	3	0.44 (0.09–2.20)	3	0.33 (0.07–1.55)
Tertile 3 (>44.5 U/T g protein)	11	1.48 (0.56–3.94)	8	1.68 (0.50–5.74)	9	0.98 (0.31–3.14)
*P for trend* **		*0.43*		*0.34*		*0.82*
**GPX3 activity**						
Per 10 U/L	27	0.88 (0.61–1.28)	16	0.83 (0.50–1.39)	17	0.70 (0.42–1.18)
Tertile 1 (<674 U/L)	8	reference	4	reference	7	reference
Tertile 2 (674–787 U/L)	11	1.86 (0.62–5.57)	8	1.89 (0.48–7.44)	6	1.17 (0.32–4.24)
Tertile 3 (>787 U/L)	8	1.52 (0.52–4.47)	4	1.37 (0.29–6.40)	4	0.85 (0.22–3.34)
*P for trend* **		*0.48*		*0.70*		*0.83*
**SEPP1 concentration**						
Per µg/L	27	1.46 (1.05–2.05)	16	1.31 (0.84–2.02)	17	0.85 (0.51–1.42)
Tertile 1 (<5.4 µg/L)	7	reference	2	reference	10	reference
Tertile 2 (5.4–6.1 µg/L)	6	1.89 (0.58–6.13)	5	4.08 (0.70–23.69)	2	0.22 (0.04–1.12)
Tertile 3 (>6.1 µg/L)	14	3.95 (1.42–10.97)	9	6.53 (1.31–32.58)	5	0.67 (0.21–2.19)
*P for trend* **		***0.006***		***0.02***		*0.48*
**MDA**						
Per 0.1 µmol/L	27	1.10 (0.97–1.24)	16	1.13 (0.98–1.29)	17	1.04 (0.87–1.24)
Tertile 1 (<0.751 µmol/L)	5	reference	4	reference	5	reference
Tertile 2 (0.751–0.971 µmol/L)	13	2.80 (0.95–8.21)	7	1.29 (0.33–4.97)	8	2.05 (0.62–6.80)
Tertile 3 (>0.971 µmol/L)	9	2.04 (0.67–6.27)	5	1.14 (0.28–4.62)	4	0.71 (0.18–2.81)
*P for trend* **		*0.33*		*0.91*		*0.54*
**PCC**						
Per 0.1 nmol/mg protein	27	1.21 (0.60–2.42)	16	0.64 (0.25–1.65)	17	1.19 (0.46–3.08)
Tertile 1 (<0.333 nmol/mg protein)	8	reference	8	reference	7	reference
Tertile 2 (0.333–0.384 nmol/mg protein)	8	1.11 (0.40–3.10)	4	0.43 (0.12–1.56)	5	0.75 (0.22–2.55)
Tertile 3 (>0.384 nmol/mg protein)	11	1.38 (0.55–3.49)	4	0.45 (0.13–1.56)	5	0.62 (0.19–2.08)
*P for trend* **		*0.49*		*0.18*		*0.44*

* HRs were adjusted for age at time of blood draw (5 categories), waist: hip ratio (quartiles), sex, smoking status, NSAID use (both for never, former or current) and serum selenium concentrations (continuous).

** P for trend was obtained by assigning median values of each tertile.

Two SNPs in the *GPX3* gene were statistically significantly associated with the risk of EA (P for trend = 0.03 for rs4958872 and P for trend = 0.04 for rs3792797); however, the overall genetic variation was not significant (global P = 0.33) (**Table 4**). None of the other SNPs in selenoenzyme genes, when combined within a gene and assessed individually, was associated with the risk of neoplastic progression to EA (global P = 0.12–0.69; P for trend = 0.08–0.99).

**Table 4:** Association between SNPs in Selenoenzymes and Risk of Neoplastic Progression to Esophageal Adenocarcinoma*.

		Esophageal adenocarcinoma		Aneuploidy		Tetraploidy	
Gene	SNP	HR (95% CI)	P trend	HR (95% CI)	P trend	HR (95% CI)	P trend
*GPX1*	rs3448	0.79 (0.39–1.60)	0.51	1.95 (0.71–5.35)	0.20	0.98 (0.37–2.58)	0.96
	rs1987628	0.56 (0.30–1.19)	0.14	0.62 (0.24–1.59)	0.32	1.61 (0.72–3.58)	0.25
	Global P	0.12		0.37		0.48	
*GPX2*	rs4902347	1.45 (0.66–3.21)	0.36	1.21 (0.39–3.75)	0.74	1.48 (0.47–4.70)	0.50
	rs4902346	1.37 (0.71–2.63)	0.35	1.18 (0.46–3.03)	0.73	1.72 (0.74–3.98)	0.21
	rs2071566	1.05 (0.58–1.92)	0.87	0.89 (0.38–2.07)	0.79	1.44 (0.74–2.79)	0.23
	rs10121	0.82 (0.27–2.50)	0.73	0.91 (0.21–3.93)	0.89	1.62 (0.51–5.15)	0.41
	Global P	0.17		0.26		0.60	
*GPX3*	rs3763013	1.34 (0.76–2.34)	0.31	0.95 (0.41–2.19)	0.90	1.25 (0.61–2.55)	0.54
	rs3805435	1.14 (0.34–3.85)	0.84	1.24 (0.29–5.30)	0.77	0.35 (0.04–3.01)	0.34
	rs8177406	0.77 (0.34–1.75)	0.54	1.09 (0.40–2.98)	0.86	1.06 (0.44–2.55)	0.90
	rs4958872	2.08 (1.07–4.05)	0.03	1.00 (0.39–2.56)	0.99	0.60 (0.21–1.71)	0.34
	rs736775	1.47 (0.76–2.75)	0.22	1.00 (0.43–2.32)	0.99	0.77 (0.35–1.69)	0.52
	rs3792797	2.22 (1.04–4.76)	0.04	1.30 (0.45–3.77)	0.62	0.69 (0.21–2.27)	0.54
	Global P	0.33		0.65		0.69	
*GPX4*	rs8178974	0.83 (0.37–1.85)	0.65	0.46 (0.14–1.51)	0.20	0.90 (0.29–2.76)	0.85
	rs8178977	1.71 (0.91–3.21)	0.10	1.71 (0.68–4.33)	0.26	0.41 (0.14–1.19)	0.10
	rs713041	0.69 (0.40–1.20)	0.19	1.32 (0.61–2.86)	0.48	1.98 (0.93–4.23)	0.08
	rs2074451	0.66 (0.38–1.15)	0.14	1.47 (0.64–3.40)	0.37	1.84 (0.85–3.97)	0.12
	Global P	0.46		0.45		0.34	
*SEPP1*	rs11959466	0.58 (0.15–2.24)	0.43	0.70 (0.19–2.58)	0.59	0.47 (0.06–3.67)	0.47
	rs12055266	0.86 (0.46–1.61)	0.63	0.94 (0.43–2.06)	0.88	1.06 (0.50–2.28)	0.87
	rs3797310	0.97 (0.53–1.77)	0.91	1.24 (0.59–2.62)	0.57	1.03 (0.48–2.21)	0.93
	rs230819	1.03 (0.61–1.74)	0.93	1.12 (0.58–2.16)	0.73	0.68 (0.34–1.34)	0.26
	rs13168440	1.02 (0.50–2.09)	0.95	0.75 (0.27–2.09)	0.58	0.38 (0.11–1.35)	0.14
	rs3877899	1.34 (0.71–2.51)	0.37	0.75 (0.29–1.95)	0.56	0.47 (0.16–1.39)	0.17
	Global P	0.65		0.14		0.46	

* HR and 95% CIs were based on additive model and adjusted for age at baseline (5 categories), waist: hip ratio (quartiles), sex, smoking status, NSAID use and Caucasian ethnicity; Global p is based on the log likelihood ratio statistics comparing the model with and without all SNPs in a given gene.

## Discussion

Our study found no evidence of an association of serum selenium concentration with the risk of neoplastic progression to EA, aneuploidy or tetraploidy. However, SEPP1 concentration was strongly and significantly positively associated with risk of EA and aneuploidy, though not tetraploidy. None of the overall variation in *GPX1-4* and *SEPP1* genes was significantly associated with the risk of neoplastic progression to EA.

Our null finding on serum selenium is consistent with the case-cohort study in The Netherlands that observed no association between toenail selenium and the risk of progression from BE to EA or high-grade dysplasia [28]. However, our current prospective analysis did not replicate findings from our previous cross-sectional analysis that showed an inverse association of serum selenium [>1.5 µM (equivalent to 118 µg/L)] with aneuploidy, high-grade dysplasia and 17p loss of heterozygosity, all measures of neoplastic progression to EA [10]. This may be partly explained by the different outcomes investigated in the two analyses and by prevalent and incident cases. Further, our sensitivity analysis excluding participants who had high-grade dysplasia or aneuploidy and/or tetraploidy at baseline did not differ from the overall finding, suggesting that the prevalent condition did not affect our null finding. Hence, an inverse association observed in our previous cross-sectional analysis is most likely due to reverse causality or the fact that high serum selenium in our cohort might be associated with other factors that increase the risk of neoplastic progression to EA.

Within the general population, clinical trials of selenium supplementation with or without other micronutrients in China and the U.S. reported non-significant 6% to 70% decreased risk of esophageal cancer with supplementation, although their 95% CI were large, likely due to their small sample sizes [1], [2], [5]. Two small case-control studies and a case-cohort of the trial in China, which reported a significant 44% lower risk of esophageal cancer with low selenium concentration (>82 µg/L vs. <60 µg/L), also support a protective effect of serum selenium on esophageal cancer [4], [6], [7]. In addition, a recent case-cohort study in The Netherlands reported a significant inverse association between baseline toenail selenium and the risk of EA among women and never smokers, but not among all participants [29]. The discrepancy between our results and findings from these trials and observational studies may be explained in part by the difference in selenium status and the common subtype of esophageal cancer in each study population. Selenium concentrations were lower in those populations [1], [2], [4], [5] than those in our cohort; the mean serum selenium concentration ranged from 72 to 116 µg/L in the trials [4], [5] and observational studies [6], [7] and the median toenail selenium concentration in the case-cohort study in The Netherlands was 0.55 µg/g [29] and was substantially lower than in U.S. populations (e.g., 0.84 [30] and 1.52 µg/g [31]). By contrast, our population was selenium-replete (mean = 135 µg/L and range = 67 to 213 µg/L) and included only five participants with serum concentrations below 90 µg/L, the proposed threshold of the antioxidant activity for GPX [32], [33]. Accordingly, in our cohort, selenium concentrations may be in a range where higher concentrations have no further benefit. In addition, the difference in the subtype of esophageal cancer needs to be addressed since risk estimates were not reported separately by subtype in trials in China [1], [3], [4], which most likely would have included predominantly squamous cell carcinoma given the high prevalence of this subtype in the area. By contrast, the outcome in our study was EA. Risk profiles for these two subtypes differ substantially [34], which suggests different etiologies between the two cancer subtypes and may also have contributed to the discrepant finding between our study and trials in China.

Few studies have investigated associations of selenoenzyme activity or selenoprotein concentrations with cancer [35]–[39] or BE risk [40]. Lower GPX1 activity was observed in prostate cancer cases than controls [36], while GPX1 activity was not associated with the risk of colorectal [35] or breast cancer [37]. In two previous studies that compared human esophageal tissue samples, higher GPX2 expression and lower GPX3 expression were observed in BE patients than that in healthy controls [40] and the expression of GPX3 was lost in EA patients [39].

To our knowledge, our study is the first to report a positive association of SEPP1 concentrations with the risk of progression from BE to EA and aneuploidy. Aside from being a carrier of selenocysteines, SEPP1 itself has antioxidant properties [41], [42] and we hypothesized that SEPP1 would be associated with a lower risk of neoplastic progression to EA, which is in contrast to our finding. Nonetheless, SEPP1 was positively associated with C-reactive protein concentrations [43] and as a peroxynitrite scavenger, could be induced by elevated peroxinitrite (ONOO^−^) [44], especially at the relatively high selenium status of our cohort. Peroxynitrite and its precursors, superoxide (O_2_^− •^) and nitric oxide (NO) [45], [46], have been hypothesized to induce the progression to BE and EA [45], [47], [48] and it is possible that SEPP1 may act as a marker of elevated peroxynitrite production in aneuploidy and EA. However, there was no cross-sectional association between SEPP1 concentrations and aneuploidy in our study. The fact that such an association would have been expected to be stronger than a prospective association does not support the hypothesis that SEPP1 is a peroxynitrite scavenger in neoplastic progression of BE to EA. Hence, we cannot rule out the possibility of a chance finding.

Only a single previous study has investigated the association between two potentially functional candidate variants in the *GPX2* gene (rs4902346 and rs2737844, also known as gastrointestinal GPX) and the risk of EA in a case-control study; however, no association was found [8]. Consistent with that finding, our study did not find an association of *GPX2* with the risk of neoplastic progression to EA, nor did we find such an association with *GPX1*, *GPX 4* and *SEPP1* genes. Although two *GPX3* variants were individually significantly associated with the risk of EA, the overall variation was not significant. Hence, this finding may be due to chance. Our study was limited in sample size; in our post-hoc power calculation while adjusting for alpha level to 0.0114 to account for multiple comparisons of SNPs per gene, the powers of detecting the risk estimate of 1.20 to 1.50 ranged from 1.4% to 3.2% for the observed minor allele frequency of 5% and from 2.4% to 11.9% for the observed minor allele frequency of 49%, respectively for all three measures of the risk of neoplastic progression. Hence, our study was almost certainly underpowered to detect the type of weak associations found in genome-wide scans for other cancers.

Strengths of our study include the prospective design, the long follow-up (on average 7.3 years) and the high frequency of follow-up visits (on average 5.7 visits) for biospecimen collection. We were also able to evaluate flow cytometric abnormalities (i.e., aneuploidy and tetraploidy) that reflect neoplastic progression, which extended our ability to measure progression. Detailed exposure assessments allowed us to adjust for important potential confounding in our analysis. Finally, we extended the evaluation of common variants in selenoenzyme/selenoprotein genes by including more genes than in a previous study of EA [8].

One very important limitation of our study is the relatively small number of endpoints in our cohort. To some extent, this is mitigated by the involvement of high-risk participants and the use of valuable intermediate markers of neoplastic progression. Our ability to detect an association between selenium and the risk of neoplastic progression to EA also may have been limited by the relatively high selenium concentrations in our cohort. We used a single serum selenium measurement, which may not capture participants' selenium intake during the entire follow-up period. Finally, serum selenium concentrations may not reflect tissue concentrations, which may be the exposure of most importance.

In summary, we found no evidence of association of selenium concentrations with the risk of neoplastic progression to EA. This finding is inconsistent with our previous cross-sectional analysis and suggests that findings from cross-sectional studies of selenium and neoplastic progression need to be interpreted with caution. Our study is the first to observe positive associations of plasma SEPP1 concentrations with the risk of neoplastic progression to EA, a finding that warrants further investigation.
